# Ethereal Solution of Gun Cotton

**Published:** 1848-04

**Authors:** 


*Ethereal Solution of Gun Cotton.*—This new *Boston notion* bids fair to compete for celebrity with its predecessor, the Letheon. From the fact that the advertising sheet of the Boston Journal is filled with advertisements for the sale of the new article, we infer that there is a brisk demand for it at Boston. We commend to the attention of our readers the communication in the eclectic department of this No., on the subject. Mr. Bigelow, we are informed, is a medical student. The honor of the discovery is contested by Mr. John P. Maynard, a fellow student of Mr. Bigelow. We trust the parentage of the new discovery will be settled with less discussion and ink-shed, than in the instance of the Letheon.

